# Targeting microRNA to improve diagnostic and therapeutic approaches for malignant mesothelioma

**DOI:** 10.18632/oncotarget.20409

**Published:** 2017-08-24

**Authors:** Kimberly A. Birnie, Cecilia M. Prêle, Philip J. Thompson, Bahareh Badrian, Steven E. Mutsaers

**Affiliations:** ^1^ Institute for Respiratory Health, Centre for Respiratory Health, Harry Perkins Institute of Medical Research, QEII Medical Centre, School of Biomedical Sciences, University of Western Australia, Perth, Western Australia, Australia; ^2^ Centre for Cell Therapy and Regenerative Medicine, Harry Perkins Institute of Medical Research, QEII Medical Centre, School of Biomedical Sciences, University of Western Australia, Perth, Western Australia, Australia

**Keywords:** malignant pleural mesothelioma, malignant peritoneal mesothelioma, microRNA, biomarkers, therapies

## Abstract

Malignant mesothelioma is an aggressive and often fatal cancer associated with asbestos exposure. The disease originates in the mesothelial lining of the serosal cavities, most commonly affecting the pleura. Survival rates are low as diagnosis often occurs at an advanced stage and current treatments are limited. Identifying new diagnostic and therapeutic targets for mesothelioma remains a priority, particularly for the new wave of victims exposed to asbestos through do-it-yourself renovations and in countries where asbestos is still mined and used. Recent advances have demonstrated a biological role for the small but powerful gene regulators microRNA (miRNA) in mesothelioma. A number of potential therapeutic targets have been identified. MiRNA have also become popular as potential biomarkers for mesothelioma due to their stable expression in bodily fluid and tissues. In this review, we highlight the current challenges associated with the diagnosis and treatment of mesothelioma and discuss how targeting miRNA may improve diagnostic, prognostic and therapeutic approaches.

## INTRODUCTION

The mesothelium is a monolayer of cells extending over the surface of the serosal cavities and organs. This layer facilitates free movement between tissues and organs whilst protecting them from infection and injury [1–3]. Mesothelioma is an aggressive cancer that develops in the mesothelium and is strongly associated with asbestos exposure [4]. The fibrous mineral erionite [5], carbon nanotubes [6, 7], genetic mutations [8], radiation [9] and Simian Virus 40 [10] have also been linked to mesothelioma.

The global incidence of mesothelioma has increased over the last decade and is predicted to peak sometime before 2030 [11]. Countries with the highest rates of mesothelioma include the USA and the UK, however Australia and Italy rank highly per capita [12]. Mesothelioma has a long latency period (20–40 years) following asbestos exposure [11]. Therefore, the new wave of predicted victims exposed through building demolition, renovation and repair [13] and in countries where asbestos is still mined and used (India, Vietnam, China, Russia, Zambia, Colombia and Kazakhstan) [14, 15], will likely see mesothelioma be a health burden beyond 2030.

Mesothelioma has a poor prognosis with a median survival of 4 to 14 months [12, 16]. Survival is influenced by histological subtype (epithelioid, biphasic, sarcomatoid) as epithelioid patients survive on average 9 months longer than sarcomatoid patients [12, 17, 18]. Cases of malignant pleural mesothelioma (MPM) are most commonly reported (80%) followed by malignant peritoneal mesothelioma (PMM) (20%) and rarely, mesothelioma of the pericardium [19–21] and testis [22].

### Malignant pleural mesothelioma (MPM)

MPM consists of small tumour nodules that extend along the pleural surface. These tumours eventually enclose and invade the lung [15, 23]. Symptoms of MPM include chest tightness, pain and shortness of breath [24]. In 90% of patients, these symptoms will be caused by the presence of a pleural effusion (PE) [25].

PE, pleural thickening and pleural nodules are likely to be evident upon imaging of suspected cases [26, 27], however these findings do not distinguish MPM from other metastatic diseases [26, 28]. A cytological diagnosis of epithelioid MPM using PE samples is possible [29]. However, sarcomatoid cells are not often shed into the pleural space, therefore tissue demonstrating the malignant characteristics of invasion, cellular atypia and necrosis is required [30]. Collecting tissue by biopsy is invasive and can complicate disease management through the potential seeding of tumour cells [31]. To identify ‘at risk’ individuals and patients with early stage MPM, serum and PE markers have been investigated. The diagnostic accuracies reported for the more promising biomarkers mesothelin (MSLN) [32, 33], osteopontin [34, 35] and fibulin-3 [36, 37] are variable [31]. Therefore, there are currently no biomarkers that can be used alone for the accurate diagnosis of MPM.

Treatment of MPM requires multiple therapeutic modalities. Surgery (pleurectomy with or without decortication (PD) and extrapleural pneumonectomy (EPP)) can improve symptoms but rarely eradicate microscopic disease [38]. The regimen considered to be the standard of care for the palliation of MPM is the combination of pemetrexed with cisplatin [39, 40]. Compared to cisplatin alone, this combination improves patient survival by a few months [40]. Radiotherapy can also be given for palliative reasons [16, 41] but rarely improves survival outcomes [26, 42].

No new treatments for MPM exist outside the clinical trial setting [38, 39]. However, novel targets such as growth factors, apoptotic signalling pathways and various aspects of the immune system are being investigated. The immune checkpoint inhibitors cytotoxic T-lymphocyte-associated antigen 4 (CTLA-4) and programmed death 1 (PD-1) have become popular targets in recent times. Novel drugs targeting CTLA-4 and PD-1 have been approved for the treatment of melanoma and non-small cell lung cancer (NSCLC) and are being tested in ongoing trials for many cancers including MPM [39]. Other therapeutic options being investigated include the administration of oncolytic viruses, vaccination strategies to induce antigen-specific cell-mediated immune responses [43] and restoring down-regulated miRNA [44].

### Malignant peritoneal mesothelioma (PMM)

PMM is often found as a diffuse tumour of the intestinal serosa or a large mass on the omentum or mesentery [23]. PMM presents with non-specific symptoms such as loss of appetite, nausea, vomiting, diarrhoea, constipation and ascites. Small bowel obstruction is a late feature [16].

Radiological findings that give the best indication of PMM include ascites associated with minimal soft tissue masses and preserved normal anatomy of the bowel and its mesentery [45, 46]. Laparotomy and laparoscopy with biopsy are the main diagnostic approaches used, however to ensure diagnostic accuracy, tissue samples need to be of adequate amount and quality [16]. Serum biomarkers for the less-invasive diagnosis of PMM have been investigated and potential targets include hyaluronic acid, osteopontin, mesothelin [16] and high mobility group box 1 (HMGB1) [47]. The identification of a robust biomarker for PMM is important as PMM has a high misdiagnosis rate and can easily be confused with ovarian cancer [48], diseases that affect the colon [49] and tuberculosis peritonitis [50].

The standard regimen used to treat PMM includes cytoreductive surgery (CRS) with hyperthermic intraperitoneal chemotherapy (HIPEC) using mitomycin C or cisplatin [16]. However, CRS plus HIPEC is not suitable for all patients, particularly those with disease outside the peritoneum or with poor health [51]. Whether radiotherapy improves patient survival remains unclear and adverse side effects such as adhesions and intestinal obstruction reduce the popularity of its use [16]. There are few clinical trials investigating novel avenues for the treatment of PMM, therefore new therapeutic targets are required.

Diagnosing mesothelioma can be challenging, invasive and lengthy. There is no way of identifying ‘at risk’ individuals who may benefit from early intervention. Current treatments are rarely curative and the lack of positive clinical trial results is a concern. Mesothelioma is likely to continue being a global health burden as the threat of asbestos exposure continues. If survival rates are to improve, novel treatment and diagnostic approaches are urgently needed.

## MICRORNA

**Figure 1:**
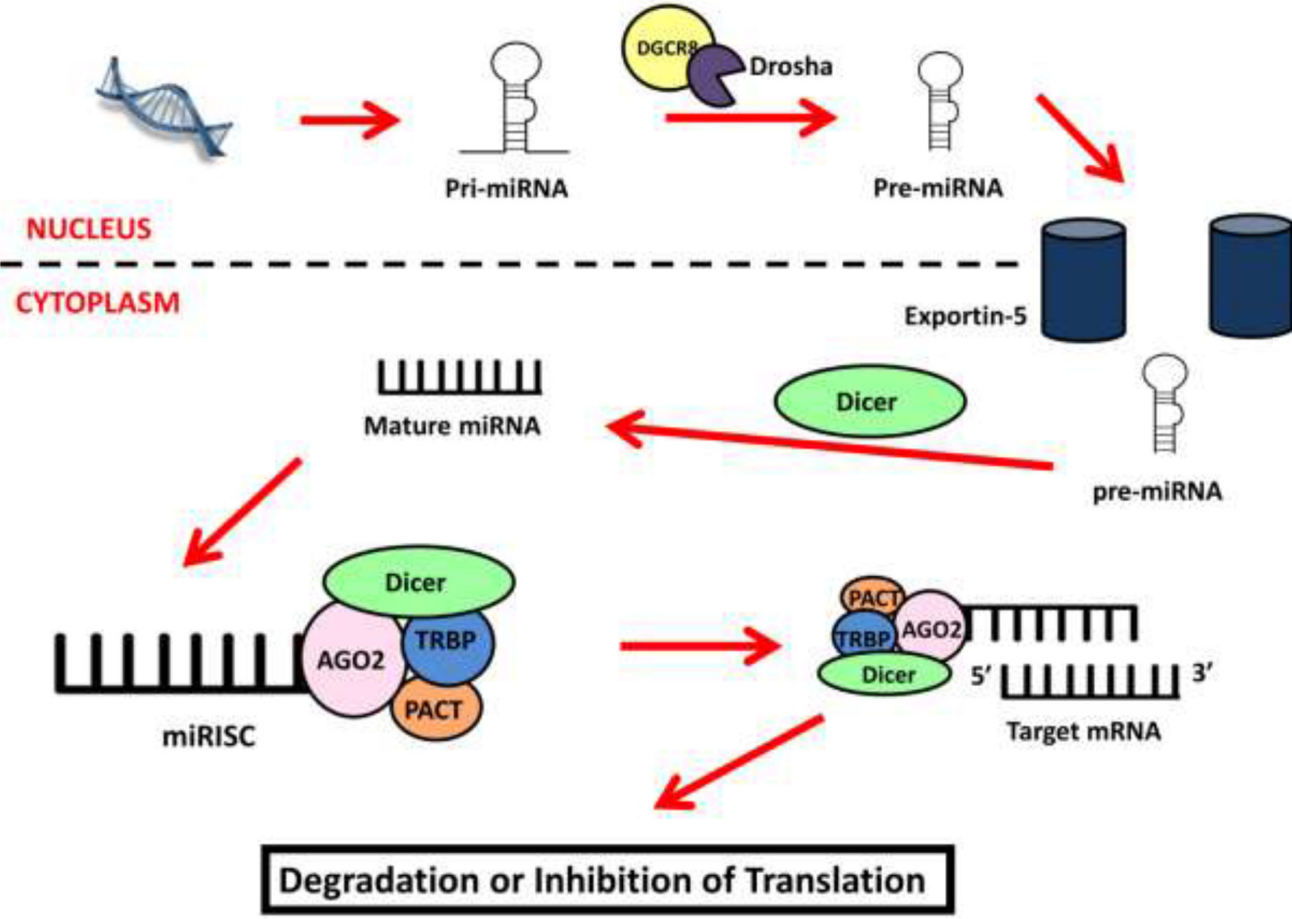
miRNA biogenesis MiRNA biogenesis starts in the nucleus where the miRNA gene is transcribed into a primary miRNA transcript (pri-miRNA) and processed by Drosha/DGCR8 into the miRNA precursor (pre-miRNA). The precursor is exported into the cytoplasm by Exportin-5 where it is cleaved by Dicer to become the mature miRNA. This strand forms the miRISC complex with the AGO2, TRBP, PACT and Dicer proteins. The miRISC uses the miRNA as a guide to identify and bind to target mRNA causing the inhibition of target genes by inducing mRNA degradation or inhibiting translation.

During the search for novel therapeutic and diagnostic targets for mesothelioma, the small but powerful gene regulators microRNA (miRNA) have become of interest. Cells produce miRNA in a multi-step process beginning in the nucleus (Figure 1) [52–55] where miRNA transcripts are cleaved by Drosha/DGCR8 to produce an intermediate structure of 60–70 nucleotides (nt) [52, 53, 56]. This precursor is transported into the cytoplasm by the Exportin-5 protein [53] where it is cleaved to form a duplex (21 to 25 nt) consisting of the mature miRNA and its complementary strand [57]. MiRNA associate with the argonaute protein to form the core of the miRNA Induced Silencing Complex (miRISC) [58] and guide the miRISC to complementary sites within target messenger RNA (mRNA) [52]. Once bound, the miRISC inhibits gene expression by initiating mRNA degradation and/or repressing translation [54, 59]. Cells can also produce miRNA through a number of non-canonical processes independent of Drosha and/or Dicer activity [60].

MiRNA have extensive regulatory potential and the imprecise base pairing between miRNA and their targets allow miRNA to regulate a multitude of genes [61, 62]. Deviations in this regulation contribute to aberrant gene expression and influence disease biology [63]. MiRNA can function as oncogenes and/or tumour suppressors and are therefore potential targets for the development of novel treatments for cancer. MiRNA also have characteristics that make them attractive biomarkers including being stably expressed in tissue and fluid [64] and being easily measurable using techniques such as quantitative real-time PCR (qPCR) [65].

## MIRNA IN MPM

### Role of miRNA in MPM biology

MiRNA were first suggested to have biological roles in MPM by Guled and colleagues in 2009. The authors identified a number of miRNA that were expressed significantly different between MPM tissue and normal pericardium and the three MPM subtypes. The miRNA were predicted to target some of the more commonly affected genes in MPM including cyclin-dependent kinase inhibitor 2A (CDNK2A), neurofibromatosis type 2 (NF2), jun oncogene, hepatocyte growth factor and platelet derived growth factor. The miRNA were also located in chromosomal areas known to be deleted or gained in MPM [66]. A number of miRNA have now been identified as aberrantly expressed in MPM with a select few shown to regulate cell activity. Those with functional roles in MPM are described below and summarised in Table 1.

**Table 1:** MiRNA with biological roles in mesothelioma

miRNA	Ref	Expression in mesothelioma vs controls	Samples Analysed	MiRNA genomic location	Chromosomal aberration in mesothelioma?	Target gene regulated in mesothelioma	Mesothelioma cell function regulated
**MPM**							
29c-5p	[67]	Lower	8 MPM cell lines, LP9 mesothelial cells	1q32.2	Yes [121]	DNMT1, DNMT3A	Proliferation, migration, invasion, colony formation, methylation
31	[68]	Lower	8 MPM cell lines, LP9, primary mesothelial culture, Met-5A	9p21.3	Yes [121, 122]	PPP6C	Proliferation, migration, invasion, colony formation
let-7a	[72, 73]	N/A	2 EphrinA1 treated MPM cell lines	22q13.31	Yes [121]	RAS oncogenes	Proliferation, migration
let-7b	[74]	N/A	2 Ursolic Acid treated MPM cell lines	22q13.31	Yes [121]	Twist	Apoptosis, EMT
16	[75, 76]	Lower	60 FFPE MPM tissues (46 Ep, 14 Bi), 23 FFPE normal pleura tissues, 6 MPM cell lines, Met-5A	13q14.2	Yes [121]	CCDN1, BCL-2, PD-L1	Proliferation, chemoresistance
34 b/c	[77, 78]	Lower	47 MPM tumours (32 Ep, 10 Bi, 4 Sa, 1 lymphohistiocytic), 10 non-neoplastic pleura, 6 MPM cell lines, 2 primary mesothelial cultures	11q23.1	Yes [121]	BCL-2	Proliferation, migration, invasion, resistance to radiotherapy
126	[80]	Lower	29 FFPE MPM tissues, 5 MPM diagnostic biopsies, 14 matched non-neoplastic tissues, 5 pneumothorax benign reactive mesothelial tissues	9q34.3	Yes [121]	IRS1, PDK, ACL	Mitochondrial metabolism, proliferation, autophagic flux
1	[83, 84]	Lower	25 MPM tumours, 25 unmatched normal pleura tissue, 7 MPM cell lines	20q13.33	Yes [121]	PIM1	Proliferation, apoptosis, migration, invasion
145	[86]	Lower	71 MPM tumours, 12 mesothelial benign cysts, 50 normal tissues, 3 MPM cell lines, primary mesothelial cell culture	5q32	Yes [123]	OCT4	Cell viability, clonogenicity, migration
21-5p	[89]	N/A	N/A	17q23.1	Yes [121]	MSLN	Proliferation
223	[90]	Lower	8 MPM cell lines (5 human, 3 mouse), primary mesothelial cell cultures, 26 MPM pleural effusions, 10 benign pleural effusions, 17 FFPE MPM tissues, 6 FFPE normal pleura	Xq12	No	STMN1	Migration
302b	[95]	N/A	2 EphrinA1 treated MPM cell lines	4q25	Yes [121]	MCL-1	Proliferation, apoptosis
193a-3p	[96]	Lower	120 MPM tissues (59 extrapleural pneumonectomy & 61 pleurectomy + decortication), 23 normal pleura, 10 MPM cell lines, Met-5A	7q11.2	Yes [124]	MCL-1	Proliferation, apoptosis, necrosis
17-5p	[97]	Lower	60 FFPE MPM tissues, 23 normal pleura, 7 MPM cell lines, Met-5A	13q31.3	Yes [121]	KCNMA1	Migration
205	[99]	N/A	74 MPM tissues (21 Bi, 18 Sa, 35 Ep)	1q32.3	Yes [121]	ZEB1, ZEB2	EMT, invasion, migration
PMM							
34a	[117]	Lower	45 PMM tissues, 5 PMM cell lines, 7 normal peritoneum	1p36.22	Yes [125]	c-MET, AKT	Proliferation, apoptosis, invasion

Ep – epithelioid, Bi – biphasic, Sa – sarcomatoid, N/A – not available, FFPE – formalin fixed paraffin embedded.

Pass and colleagues were the first to identify a miRNA with a functional role in MPM after miR-29c-5p was observed as downregulated in MPM cell lines compared to normal mesothelial controls. Transfection of the miR-29c-5p mimic in two MPM cell lines restored miR-29c-5p expression and caused an inhibition of cell proliferation, migration, invasion and colony formation. MiR-29c-5p was also suggested as a potential mediator of methylation in MPM after the expression of the DNA methyltransferases DNMT1 and DMT3A were reduced following miR-29c-5p overexpression. This study revealed that miR-29c-5p may be a tumour suppressor in MPM and is thus a potential therapeutic target [67].

MiR-31 is another downregulated miRNA in MPM caused by co-deletion of the miR-31 and CDKN2A genes from chromosome 9p21 [68]. Re-introducing miR-31 in MPM cells inhibited proliferation, migration, invasion and colony formation and reduced levels of protein phosphatase 6 (PPP6C). Aberrant PPP6C activity is associated with resistance to chemotherapy [69] and radiotherapy [70]. Therefore, loss of miR-31 in MPM may contribute to elevated PPP6C and promote chemoresistance. If this is true, miR-31 replacement therapy may be an effective tool to enhance tumour responses to chemotherapy.

Let-7 is a well-studied miRNA with a range of functions in cancer [71]. In MPM cell lines, let-7a was upregulated following activation of the Ephrin type A receptor by its ligand Ephrin A1. In turn, RAS family proto-oncogenes were suppressed causing an inhibitory effect on MPM cell growth [72]. In a subsequent study, nanoparticles packaged with let-7a inhibited MPM cell proliferation, migration and tumour growth [73]. Recently, another let-7 family member, let-7b, was shown to enhance the anti-tumour effect of ursolic acid in MPM cell lines. The overexpression of let-7b caused the cleavage of caspase-3 and PARP, the suppression of pAKT, B-catenin and Twist and the accumulation of cells in the sub-G1 phase of the cell cycle. The inhibition of let-7b in the same cell lines blocked the cytotoxicity of ursolic acid treatment and together, these results suggest that let-7b may regulate apoptosis and inhibit epithelial to mesenchymal transition (EMT) during ursolic acid treatment of MPM [74]. Increasing let-7a and b may be effective treatment strategies for MPM, either alone or in combination with novel chemotherapy agents.

MiRNA MPM therapeutic studies have recently advanced to clinical trial. MiR-16 is now the focus of a phase I trial that will be discussed in the following section of this review. The trial is based on work by Reid and colleagues who reported the downregulation of miR-15/16 in MPM tissue and cell lines in 2013. Reduced miR-15/16 was associated with increased levels of the target oncogenes CCND1 and Bcl-2 and re-expressing the miRNA in cell lines inhibited cell growth. Cell growth was inhibited most effectively following transfection of the miR-16 mimic. Restoring miR-16 also re-sensitised MPM cells to pemetrexed and gemcitabine and the intravenous administration of miR-16 in nanocells with epidermal growth factor receptor (EGFR) specific antibodies, inhibited tumour growth in mice [75]. The same laboratory has recently published results demonstrating that miR-16 is also a regulator of programmed death ligand 1 (PD-L1) in MPM and may therefore contribute to immune system evasion [76].

The miR-34 family are attractive targets for replacement therapy in MPM. The absence of miR-34b/c in MPM cells is caused by methylation and restoring miR-34b/c reverses malignant features such as migration, invasion, motility [77] and resistance to radiotherapy [78]. In normal mesothelial cells, reducing miR-34 induced cell proliferation, migration and invasion by up-regulating oncogenes such as C-MET and Bcl-2 [79]. These studies suggest that miR-34 has a role in the oncogenic transformation of mesothelial cells and the ongoing regulation of MPM biology.

Like miR-34, the expression of miR-126 in MPM is regulated by methylation [80] and also oxidative stress [81]. During such stress, miR-126 influences metabolic processes by altering mitochondrial function and inhibits malignant features such as cell growth, soft agar colony formation and tumour formation in mice [81]. MiR-126 potentially inhibits tumour progression through its ability to induce autophagic flux, thus supporting the idea that increased levels of autophagy may be protective in MPM [82].

MiR-1 was identified as a potential tumour suppressing miRNA in MPM when miR-1 was observed as downregulated in tumour samples compared to normal pleural mesothelium. Overexpressing miR-1 in MPM cells inhibited proliferation and induced apoptosis [83]. In a subsequent study, miR-1 was reported as downregulated in MPM cell lines and overexpressing miR-1 in two of these lines led to an inhibition of cell growth, invasion, migration and reduced levels the proto-oncogene PIM1 [84]. Recent evidence suggests that PIM1 is overexpressed in MPM and can influence MPM cell function [85]. Therefore, both miR-31 and PIM1 are potential targets for future MPM therapeutic studies.

MiR-145 is also downregulated in MPM cell lines and tumours and restoring miR-145 reduced proliferation, migration and invasion *in vitro* and inhibited tumour growth in mice. MiR-145 potentially exerts these tumour suppressive effects by regulating the transcription factor and stem cell marker OCT4 [86]. A recent study has shown that OCT4/SOX2 may be useful markers for identifying MPM cancer stem cell populations *in vitro*. Cells with high OCT4/SOX2 levels were resistant to chemotherapy, barely affected by re-expression of the NF2 tumour suppressor, and had a high-tumour initiating capability *in vivo* [87]. It has been hypothesised that this sub-population of cells is responsible for the poor response of MPM to treatment and important for tumour relapse. The role of miR-145 in the regulation of OCT4 in this MPM cell population will be important to investigate.

Identifying miRNA target genes is an important process for understanding how miRNA regulate cell function and disease biology. This can be done using results reported from previous studies, prediction software or affinity purification approaches. The “miR-CATCH” technique involves an affinity capture oligonucleotide that is used to co-purify a single target mRNA together with all endogenously bound miRNA [88]. This technique was combined with next generation sequencing to identify miRNAs that regulate the commonly upregulated gene in MPM MSLN. MiR-21-5p was identified as a candidate regulator of MSLN which was confirmed following miR-21-5p overexpression in a panel of MPM cell lines and the transformed mesothelial cell line MET-5A. The increased expression of miR-21-5p reduced MSLN expression and inhibited MPM cell proliferation, therefore uncovering another potential tumour suppressing miRNA in MPM [89].

MiR-223 was similarly identified by our laboratory as downregulated in MPM when miR-223 levels were found to be significantly lower in MPM cell lines, tissue and cells isolated from MPM PE compared to controls [90]. One target of miR-223 that is overexpressed in MPM is stathmin (STMN1) [91]. STMN1 is highly expressed in many malignancies and reducing STMN1 consistently inhibits cell growth, motility, invasion and the formation of metastasis *in vivo*. These processes are associated with changes in cell morphology and a decrease in microtubule stability [92]. We showed that re-expressing miR-223 in two MPM cell lines reduced STMN1 expression and MPM cell migration. We also showed that loss of miR-223 and overexpression of STMN1 in MPM could be due to aberrant c-JUN N-terminal kinase (JNK) signalling. Therefore, a potential tumour suppressive role for the JNK-miR-223-STMN1 axis was suggested and a novel role for JNK in MPM was revealed [90]. MiR-223 along with a number of other miRNA may also regulate tumour protein 53 (TP53) in MPM. These miRNA were identified as downregulated in tumours expressing the negative regulator of TP53 MDM2 [93]. Functional studies are required to validate these findings.

Myeloid cell leukaemia-1 (Mcl-1) is an anti-apoptotic protein regulated by a number of miRNA in MPM. Mcl-1 is overexpressed in MPM and is associated with the resistance of MPM cells to apoptosis [94]. Khodayari and colleagues recently determined that Mcl-1 was downregulated in MPM cells following the upregulation of miR-302b during ephrin-A1-mediated MPM cell growth inhibition. Transfecting MPM cells with miR-302b reduced Mcl-1 expression, cell and tumoursphere growth and induced apoptosis [95]. Mcl-1 is also regulated by miR-193a-3p in MPM and transfecting MPM cells with the miR-193a-3p mimic inhibited cell growth whilst inducing apoptosis and necrosis. MiR-193a-3p, delivered to MPM tumours in nanocells with EGFR antibodies, inhibited xenograft growth and induced tumour cell apoptosis in mice [96]. Using miRNA replacement therapy to target Mcl-1 in patients may prove to be an effective treatment for MPM.

The same laboratory recently published results supporting a role for miR-17-5p in regulating MPM cell migration [97]. An integrative approach was used to compare miRNA expression data from previous studies and mRNA gene expression datasets. Amongst the top enriched miRNA was the miR-17 family that was downregulated in MPM samples and significantly associated with the epithelioid subtype. The top enriched mRNA signalling pathways included genes linked to MPM cell migration. Some of these are regulated by miR-17 including *KCNMA1* which encodes for the calcium-activated potassium channel subunit alpha 1 (KCa1.1) protein. In MPM cell lines, *KCNMA1* and KCa1.1 were downregulated along with cell migration and invasion when these cells were transfected with the miR-17-5p mimic. Targeting KCa1.1 with the inhibitor paxilline also significantly inhibited MPM cell migration and colony formation. Therefore, inhibiting KCa1.1 using either the channel blocker paxilline or miR-17-5p replacement, may serve as novel treatments for MPM.

The morphologies of the different MPM subtypes are likely due to the different EMT stages [98]. During a study to explore the role of EMT in the three histological subtypes, Fassini et al., discovered that miR-205 was expressed significantly higher in epitheliod cells and tissue compared to both the biphasic and sarcomatoid subtypes. Therefore, loss of miR-205 correlated with a mesenchymal phenotype and a more aggressive tumour [99]. MiR-205 is a known regulator of EMT and maintains an epithelial phenotype by reducing ZEB1 and 2 and enhancing E-cadherin expression [100]. Transfecting miR-205 into MPM cell lines consistently reduced ZEB1 and 2 and cell migratory capability, thus suggesting a role for miR-205 in negatively regulating malignant features in MPM [99].

Most of the miRNA described above are downregulated in MPM and serve as potential tumour suppressors. This is a common phenomenon that has been reported in many malignancies. Interestingly, the genomic locations of the miRNA genes are associated with chromosomal aberrations that have been identified in MPM tumours and cells (Table 1). Therefore, chromosomal abnormalities are likely the cause of the global downregulation of miRNA in mesothelioma.

### MiRNA replacement therapy for MPM

MiRNA are attractive therapeutic targets because of their powerful regulatory capabilities. Targeting multiple signalling pathways through a single miRNA may provide an effective way of combating drug resistance and improving tumour responses. Given that most miRNA are downregulated in MPM, strategies aimed at replacing miRNA in MPM may be therapeutically beneficial.

MiRNA replacement therapy for MPM has been an effective inhibitor of tumour growth in mice [73, 75, 81, 86, 96]. The most important development in moving this treatment forward to the clinic was the development of the miRNA delivery vehicles TargomiRs, by EnGeneIC. TargomiRs are minicells derived from asymmetric bacterial cell division that are loaded with miRNA mimics. They can be directed to malignant tissue using antibodies against specific tumour antigens [101, 102].

The TargomiRs used for the treatment of MPM in mice, loaded with the miR-16 mimic and conjugated with an anti-EGFR antibody [75], have now been intravenously administered to patients in a Phase I clinical trial MesomiR 1 (clinicaltrials.gov NCT02369198). Preliminary results indicate that this approach may have therapeutic benefit. All six patients enrolled in the initial stages completed the treatment regime. Four of the six showed stable disease and one patient had a partial response after eight weeks [44]. Ongoing trials will investigate the effects of increasing TargomiR dosage and compare TargomiR treatment to second or third-line chemotherapy.

### MiRNA as diagnostic biomarkers for MPM

The lack of successful diagnostic and prognostic markers for MPM has encouraged researchers to investigate novel targets such as miRNA (summarised in Tables 2 and 3). Early research identified the miR-200 family as potential candidates for discriminating MPM from other cancers that invade the lung such as adenocarcinoma [103, 104]. A diagnostic assay based on the expression of miR-193-3p, miR-200c and miR-192 was developed reaching a sensitivity of 100% and a specificity of 94% in a blinded validation set of 68 samples from the lung and pleura [104]. A recent study comparing miRNA profiles in MPM tissue to non-neoplastic pleura using qPCR, identified a panel of four miRNA including miR-126, miR-143, miR-145 and miR-652 as significantly downregulated in MPM. These results were validated in a larger cohort and when the four miRNA were combined using logistic regression analysis, a high diagnostic accuracy (area under the curve (AUC)), as determined by receiver operator curve (ROC) analysis, of 0.96 was achieved [105]. Despite these promising results, these studies rely on miRNA expression in tissue. Preferably, a diagnostic test would measure miRNA in samples acquired in a less invasive way such as blood or urine.

**Table 2:** Potential diagnostic miRNA for MPM

miRNA	Ref	Source	Cohort	Number	MPM Histological Subtype	Statistical Measure
200c, 141, 200b, 429	[102]	Tissue	1	15 MPM, 10 lung AD	N/A	AUC > 0.9 for each miRNA
			2	100 MPM, 32 lung AD	32 U, 39 Ep, 19 Bi, 10 Sa	
200c, 192, 193a-3p	[104]	Tissue	1	29 MPM, 140 carcinomas	22 Ep, 1 Bi, 6 Sa	sensitivity 100%specificity 94%
			2	48 MPM, 136 carcinomas	6 U, 29 Ep, 2 Bi, 7 Sa	
			3	14 MPM 49 carcinomas	8 Ep, 4 Bi, 2 Sa	
126, 143, 145, 652	[105]	Tissue	1	5 MPM, 5 matched diagnostic biopsies, 5 matched non-neoplastic pleura	5 Ep	AUC 0.96 for miRNA combined
			2	40 MPM, 12 matched diagnostic biopsies, 14 matched non-neoplastic pleura, 5 non-neoplastic reactive mesothelium	27 Ep, 25 Bi	
625-3p	[106]	Serum	1	5 MPM, 3 healthy	3 Ep, 2 Sa	AUC 0.8
			2	5 MPM, 14 healthy	1 U, 9 Ep, 3 Bi, 2 Sa	
			3	30 MPM, 10 asbestosis	1 U, 29 Ep,	
		Tissue	4	18 MPM, 7 normal pericardium	15 Ep, 3 Bi	
103	[107]	Cellular fraction of peripheral blood	1	23 MPM, 17 asbestos exposed, 25 healthy	3 U, 12 Ep, 7 Bi, 1 Sa	AUC 0.75 - 0.87
126	[108]	Tissue	1	10 MPM 5 normal mesothelium	9 Ep, 1 Sa	AUC 0.7
			2	27 MPM & adjacent normal tissue	23 Ep, 3 Bi, 1 Sa	
		Serum	3	44 MPM, 196 asbestos exposed, 50 healthy	30 Ep, 8 Bi, 6 Sa	sensitivity 60 - 73%, specificity 74%
126, 132-3p	[109]	Plasma	1	21 MPM, 21 asbestos exposed	14 Ep, 4 Bi, 3 Sa	AUC ~ 0.8 for each miRNA and combination
			2	22 MPM, 44 asbestos exposed	4U, 14 Ep, 2 Bi, 2 Sa	
197-3p, 1281, 32-3p	[110]	Serum	1	10 MPM, 10 asbestos exposed, 10 healthy	N/A	AUC ~ 0.7 for each miRNA
			2	20 MPM, 15 asbestos exposed, 14 healthy	N/A	
126, 21	[111]	Tissue	1	40 FFPE benign pleura, 51 FFPE MPM	34 Ep, 10 Bi, 75 Sa	AUC 0.92 for miRNA combination
		Archived cytology samples	2	24 Reactive mesothelium, 29 MPM	29 Ep	

U – unknown, Ep – epithelioid, Bi – biphasic, Sa – sarcomatoid, N/A – not available, FFPE – formalin fixed paraffin embedded.

**Table 3:** Potential prognostic miRNA for MPM

miRNA	Ref	Source	Cohort	Number	MPM Histological Subtype	Expression change and survival
29c-5p	[67]	Tissue	1	37 MPM	23 Ep, 14 other	Higher expression = longer survival
			2	92 MPM	58 Ep, 34 other	
17-5p, 30c	[112]	Tissue	1	24 MPM	8 Ep, 8 Bi, 8 Sa	Lower expression = longer survival in sarcomatoid MPM
31	[113]	Tissue	1	25 FFPE MPM	16 Ep, 4 Bi, 5 Sa	Lower expression = longer survival in sarcomatoid MPM
21-5p, 23a-3p, 30e-5p, 221-3p, 222-3p, 31-5p	[114]	Tissue	1	64 EPP MPM	47 Ep, 17 Bi	Signature is associated with longer survival
			2	43 PD MPM	25 Ep, 13 Bi, 5 Sa	
Let-7c-5p, 151a-5p	[115]	Tissue	1	52 FFPE MPM	43 Ep, 8 Bi, 1 Sa	Lower expression = longer survival
			2	16 fresh/frozen MPM	11 Ep, 4 Bi, 1 Sa	

Ep – epithelioid, Bi – biphasic, Sa – sarcomatoid, FFPE – formalin fixed paraffin embedded, EPP – extrapleural pneumonectomy, PD –Pleurectomy with decortication.

Based on 90 miRNA previously associated with MPM, Kirschner et al., identified miR-625-3p as differentially expressed in the serum/plasma of MPM patients compared to controls. The increased levels of miR-625-3p in two independent cohorts suggest that this miRNA may be a promising diagnostic marker. In both cohorts the AUC reported for miR-625-3p was approximately 0.8 [106]. In the same year, Weber and colleagues reported potential miRNA markers in the cellular fraction of human peripheral blood of MPM patients, asbestos exposed and healthy individuals. MiR-103 was identified as a potential biomarker that could better discriminate MPM from healthy controls (AUC – 0.87) compared to MPM from asbestos exposed individuals (AUC – 0.75) [107].

Serum miRNA have also been analysed in MPM vs asbestos exposed vs healthy individuals. Santarelli and colleagues chose to analyse miR-126 as a potential biomarker for MPM in these groups and found that miR-126 could differentiate asbestos exposed from healthy individuals with a sensitivity of 60% and specificity of 74% and from MPM with a sensitivity of 73% and specificity of 73%. When evaluated in combination with MSLN, decreasing miR-126 and increasing MSLN were indicative of a higher risk of developing MPM [108]. Combining miR-126 with a recently discovered biomarker for MPM miR-132-3p, provided a potential diagnostic signature with an accuracy much higher than the accuracies of using either miR-132-3p or miR-126 alone to discriminate MPM patients from asbestos exposed individuals. The combination of these two miRNA could distinguish MPM from asbestos exposed samples with a sensitivity and specificity of 77% and 86% respectively [109].

Three novel serum miRNA biomarkers were recently identified when Bononi and colleagues compared miRNA in serum from MPM patients, ex-workers exposed to asbestos and healthy individuals. In this study, the miRNA identified included miR-197-3p, miR-1281 and miR-32-3p. MiR-197-3p and miR-32-3p were both expressed significantly higher in MPM patients compared to both asbestos exposed ex-workers and healthy individuals. MiR-1281 was expressed significantly higher in MPM and asbestos exposed ex-workers compared to healthy individuals. These miRNA were moderately good discriminators between the three sample cohorts with diagnostic accuracies (AUC) of around 0.7 [110]. Combining the three miRNA may diagnose MPM and predict individuals at risk of developing this disease more efficiently. This is yet to be determined.

Together, these studies demonstrate the potential of measuring miRNA to diagnose MPM and identify ‘at risk’ individuals. The results are encouraging as the diagnostic accuracies for most of the miRNA signatures are equivalent to or superior than the diagnostic accuracies of the current MPM serum biomarkers mesothelin (AOC 0.7–0.9) [33], osteopontin (AOC 0.83) [35] and fibulin-3 (AOC 0.8) [36, 37]. However, larger prospective studies are required to validate these findings.

Given that most MPM patients develop a PE, it is surprising that the diagnostic potential of miRNA within PE has not been thoroughly investigated. PE are routinely drained to alleviate discomfort [25] and are ideal samples to analyse during the diagnostic process. To date there is only one study that suggests measuring miRNA in PE may assist a diagnosis of MPM. Cappallesso and colleagues compared the miRNA profiles in MPM and reactive mesothelial (RM) archived histological samples prepared from PE. A combination of miR-126 and miR-21 could complement the cytological assessment of PE to differentiate MPM from RM with a sensitivity of 86% and a specificity of 87% [111]. Whilst the results of this study are promising, a subset of only 15 miRNA previously associated with MPM were analysed. Therefore, miRNA with higher diagnostic accuracies could have been overlooked.

### MiRNA as prognostic biomarkers for MPM

The first study suggesting miRNA can be used to predict survival outcomes identified miR-29c-5p as an independent prognostic factor for time to disease progression as well as survival after surgical cytoreduction. Higher levels of miR-29c-5p predicted a more favourable prognosis [67]. Likewise, a more favourable outcome in sarcomatoid patients has been associated with reduced levels of miR-17-5p, miR-30c [112] and miR-31 [113].

Kirschner and colleagues reported a miRNA prognostic signature that could be used to predict survival outcomes in surgically resected MPM patients. A combination of six miRNA (miR-21-5p, miR-23a-3p, miR-30e-5p, miR-221-3p, miR-222-3p and miR-31-5p) provided a survival prediction accuracy of approximately 90% for MPM patients who had undergone EPP. When this signature was tested in an independent cohort of patients who had undergone palliative PD, survival was predicted at an accuracy of 72% [114]. Recently, a signature based on the expression of let-7c-5p and miR-151a-5p was identified in 52 MPM tumours as a potential tool for predicting survival. Higher levels of let-7c-5p and miR-151a-5p were associated with a poorer prognosis. This signature was validated in a second cohort of 16 fresh/frozen MPM tumours [115]. Correctly identifying patient prognosis using miRNA could allow for more intensive treatment after surgery to improve survival outcomes.

Whilst a number of miRNA have been identified as potential diagnostic and prognostic targets for MPM, there are discrepancies in the results reported between studies. Therefore, a systematic review and meta-analysis was recently undertaken to try and identify commonly reported miRNA in MPM. Because of the large differences in sample types and technologies used, comparing studies was difficult. The most consistent results were reported in blood and biopsy samples whereas cell line results varied greatly. Therefore, cell lines are best used only for functional assays [116].

Following an assessment of biomarker potential, a circulating miRNA signature based on the expression of miR-126, miR-103 and miR-625 in combination with MSLN was identified for distinguishing asbestos exposed individuals from MPM. Likewise the most consistently reported tissue specific miRNA (miR-16, miR-126, miR-143, miR-145, miR-192, miR-193, miR-200b, miR-203 and miR-652) were suggested to provide a MPM signature [116]. Validation studies are required to assess the clinical relevance of these signatures.

## MIRNA IN PMM

Until recently, there was no information on the role of miRNA in PMM. The authors of the first study to address this chose to investigate miR-34a in diffuse malignant peritoneal mesothelioma (DMPM) [117]. MiR-34a was chosen as its aberrant expression has been associated with numerous malignancies and a liposomal nanoparticle formulated synthetic miR-34 (MRX34) recently entered a phase I trial [118]. MiR-34a was analysed in 45 DMPM tissues, seven normal peritoneum samples and five DMPM cell lines by qPCR. MiR-34a was significantly downregulated in DMPM samples compared to controls. Re-expressing miR-34a in DMPM cell lines inhibited cell proliferation and induced apoptosis, although at a variable extent across the five cell lines. The inhibitory effects were suggested to be caused by miR-34a regulating c-MET and AXL signalling. The delay in the induction of apoptosis seen in some of the cell lines following miR-34a overexpression was likely due to the activation of ERK1/2 and AKT. This cytoprotective mechanism was most prominent in the MP115 cell line that was derived from the more aggressive biphasic subtype tumour. Upregulating miR-34a also inhibited cell invasion, tumour growth in xenograft and orthotopic mouse models and influenced the tumour microenvironment by impairing the secretion of angiogenic factors [117]. MiR-34a is the first miRNA identified as a potential target for miRNA replacement therapy in PMM.

## CONCLUSIONS

Mesothelioma is a fatal cancer induced by the presence of asbestos fibres. Diagnosis often occurs when the disease has reached an advanced stage and therapeutic modalities remain ineffective. Therefore, patients have a very poor prognosis and a reduced quality of life. The incidence of this disease is increasing as exposure to asbestos still occurs. A new wave of cases is a real concern.

In search of new diagnostic and therapeutic targets, mesothelioma research has evolved to include the analysis of the powerful gene regulators miRNA. A number of downregulated miRNA in mesothelioma, largely due to chromosomal aberrations, have been identified as regulators of oncogenic pathways. The re-expression of these miRNA in mesothelioma cells influences functions such as proliferation, migration, invasion, apoptosis, autophagy, methylation and chemoresistance. The development of TargomiRs is an exciting advancement in the field and a technology that can potentially be used to enhance the expression of any repressed miRNA in patients. It will be important to determine whether the replacement of multiple miRNAs or a combination of miRNAs with chemotherapy and other treatment modalities can enhance survival outcomes. There may also be an opportunity to personalise such a treatment for each patient's miRNA tumour profile.

Potential diagnostic and prognostic miRNA have also been identified for mesothelioma, however it is important to note there is often a lack of reproducibility in the results across studies. This is most likely caused by differences in study design such as sample and control selection, sample cohort size, using inappropriate controls such as transformed cell lines and analysing only a selection of miRNA. In agreement with a recent commentary by Micollucci and colleagues [119], these issues can be overcome if a standardised approach for sample collection, storage and analysis is developed in a collaborative effort with large sample cohorts. Such an effort is already underway in the lung cancer field with Marzi et al., performing a series of tests to optimise miRNA quantification in serum from a cohort of more than 1000 patients. Variables such as patient fasting, haemolysis, RNA isolation protocol and data normalisation approaches were identified and controlled for and a standardised method for the analysis of miRNA in serum was suggested [120]. With the advancement of technology and an increase in collaborative efforts, novel miRNA diagnostic and therapeutic approaches for mesothelioma can be developed.
